# European Federation of Animal Science

**DOI:** 10.1093/af/vfz034

**Published:** 2019-09-28

EAAP is the International Federation of Animal Science for Europe and the Mediterranean.

Join EAAP and become a member of the most exciting international animal science network and have access to many services that are indispensible for animal scientists worldwide.

More information about the EAAP and its activities can be found at: www.eaap.org


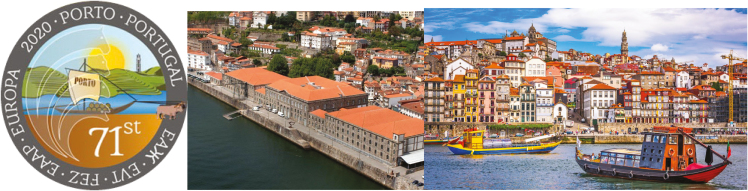


The 71^St^ Eaap Annual Meeting Will Be Held In Porto (Portugal) From 31^St^ August To 4^Th^ September 2020

After 32 years, Portugal is honored to host the annual meeting of EAAP in Porto (classified as World Heritage by UNESCO in 1996). During the meeting themes such as societal challenges of animal production in a growing world, sustainability of livestock production systems, feeding the world and ensuring resource efficiency and sustainability, technology in animal production, mountain farming systems, adaptations to climate challenges, and animal product quality and safety will be discussed. The program will cover nutrition, genetics, physiology, animal health and welfare, livestock farming systems, precision livestock farming, insect production and use, cattle, horse pig, sheep and goat production.

The EAAP Annual Meeting gives an opportunity to apply new ideas of practice through many parallel sessions, a plenary meeting, poster presentations, and discussions about scientific achievements in livestock production all around the world. It is a privileged discussion forum where the research community meets with the industry, to discuss and plan for how to address the multiple challenges that animal science sector has to cope with in the upcoming years.

All these activities make the EAAP one of the largest animal science congresses in the world.

More than 1,500 participants from more than 50 countries are expected to attend. An excellent social program will also be organized, including the welcome ceremony, an unforgettable Portuguese night, a gala dinner, technical tours, and a program for accompanying persons. The lead organizer is the Portuguese Association of Animal Science (APEZ), with the patronage of the Ministry of Agriculture, Rural Development and Fisheries from the Portuguese Government.

Agriculture in Portugal is based on small to medium-sized family-owned dispersed units

Small scale animal production in Portugal is widely related to the local breeds. Portugal has a total of 22 registered local animal breeds, most of them ancient breeds and a genetic heritage of great importance.

**Conference Information:**

http://www.eaap2020.org

**Conference local organizers** eaap2020@skyros-congressos.com

Please follow us on Facebook and Twitter

https://www.facebook.com/EAAP.ORG

https://twitter.com/eaapofficial

**INDIVIDUAL MEMBERSHIP OF EAAP**

The EAAP membership is open to all scientists. It is a great opportunity to be update on the latest publications and other relevant information in the animal sector. We have more than 3,800 members already!

Join us at http://www.eaap.org/Content/Individual_Member_Information.html

